# Acute paracetamol poisonings received at the Oran University Hospital

**DOI:** 10.1016/j.toxrep.2020.08.025

**Published:** 2020-09-07

**Authors:** Bilel Chefirat, Anissa Zergui, Chaïmaa Rahmani, Meriem Nour Belmessabih, Haciba Rezk-kallah

**Affiliations:** a Department of Pharmacy, Faculty of Medicine, University Oran1 Ahmed Ben Bella, Oran, Algeria; b Department of Pharmacology Toxicology, University Hospital of Oran, Oran, Algeria; c Environmental Health Research Laboratory, University Oran1 Ahmed Ben Bella, Oran, Algeria

**Keywords:** Paracetamol, Acute poisoning, Hepatoxicity, N-Acetylcysteine, Toxicological analysis, Rumack-Matthew nomogram

## Abstract

**Introduction:**

Paracetamol is the most commonly used drug worldwide for its analgesic/antipyretic effect and especially a non-prescription access in pharmacies. Acute Paracetamol poisoning remains problematic for clinicians because of its insidious progression to fulminant hepatitis and even death. This work proposes to draw up the epidemiological profile of acute Paracetamol poisonings.

**Materials and Methods:**

A retrospective descriptive study has been carried out over 8 years on cases of acute Paracetamol poisoning received at the Pharmacology Toxicology department of University Hospital of Oran (UHO). Data were collected using a pre-established fact sheet. Toxicological analysis was carried out by colorimetric and enzyme immunoassay method.

**Results and Discussion:**

A total of 400 cases were recorded, mainly from emergency departments of UHO (85 %). These are suicide attempts in 82 % of cases, observed especially in adolescents (69 %), and accidental poisoning in 12 % of cases, predominant in small children (89 %). Half of the patients were admitted asymptomatic in the first 24 h of intoxication. Digestive and neurological disorders were the most described (18.75 % and 20.5 %). The quantitative determination of Paracetamol showed that 16 cases had a high risk of developing liver injury and required antidote therapy, based on N-acetylcysteine. The evolution was mostly favorable (84 %) but 8 patients had liver damage and 5 deaths were recorded.

**Conclusion:**

Although it seems benign, acute Paracetamol poisoning is serious and requires adequate care making clinicians collaborate with toxicologists. The general population must be made aware of the dangers of Paracetamol. The pharmacist must provide the necessary information concerning the recommended doses and the toxicity.

## 1 Introduction

Acute poisoning is a very important reason for consultation and admission to emergency services. They constitute a real public health problem whose causes and impact must be well understood.

Paracetamol is the most consumed drug in the worldwide for its analgesic/antipyretic effect and especially a non-prescription access in pharmacies. Its toxicity is often trivialized by the general population, although it is a potent hepatotoxic in case of substantial intakes or misuse without respecting the recommended administration guidelines.

Acute Paracetamol poisoning remains very problematic for clinicians because of its insidious progression to fulminant hepatitis and even death, in the absence of adequate therapeutic management.

For this reason, we focused in this work to draw up a profile for different epidemiological, therapeutic and analytical aspects of acute Paracetamol poisoning received at University Hospital of Oran.

## 2 Materials and methods

This is a descriptive study of acute Paracetamol poisoning’ cases received at Oran University Hospital during the last eight years (2010–2017).

Data were collected prospectively, using a pre-established information sheet with information on the patient, the circumstances of poisoning, the estimated time of intoxication, the therapeutic management, etc. This sheet must accompany the biological samples for each request for toxicological analysis.

The toxicological analyzes were performed at the Pharmacology Toxicology Department for all patients even by colorimetric research of Paracetamol in the urine or by quantitative determination of Paracetamol in the blood by immunoassay (EMIT) or by both tests depending on the availability of one or the other sampling and their compliance as well as the availability of reagents.

Colorimetric research is based on acid hydrolysis of Paracetamol leading to the release of p-aminophenol. The latter is characterized after the action of phenol in the presence of ammonia resulting in the formation of a strongly colored derivative (intense royal blue).

In the performance of the EMIT Paracetamol Assay (Enzyme Multiplied Imuunoassay Test), serum is mixed with Reagent 1, which contains antibodies to Paracetamol and the coenzyme nicotinamide adenine dinucleotide (NAD). Subsequently, Reagent 2, containing Paracetamol labeled with the enzyme glucose-6-phosphate dehydrogenase (G6PDH), is added. Paracetamol in the sample and Paracetamol labeled G6PDH compete for antibody binding sites. Enzyme activity decreases upon binding to the antibody, so the Paracetamol concentration in the sample can be measured in terms of enzyme activity. Active enzyme converts oxidized NAD to NADH, resulting in an absorbance change that can be measured spectrophotometrically. This assay was performed on a V-Twin® Drug Testing System from Siemens®.

Statistical analysis was performed using SPSS version 16 (Statistical Package for Social Sciences). Qualitative variables are expressed as percentages and quantitative variables as averages.

## 3 Results and discussion

### 3.1 Profile of the intoxicated population

**Fig. 1:**
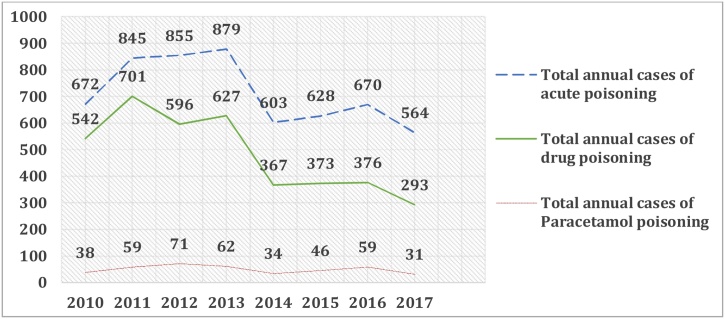
Frequency of Paracetamol in acute poisonings.

A total of 400 cases of acute Paracetamol poisoning were recorded, representing 7% of total acute poisonings and 10 % of total drug poisonings (Fig. 1).

The Department of Pharmacology Toxicology of the University Hospital of Oran is requested by several services whether intramuros or extramuros in the city of Oran and other cities in the west of the country. The cases were recorded mainly from emergency departments (medical emergencies, adult intensive care, pediatric intensive care, specialized hospitals).

**Fig. 2:**
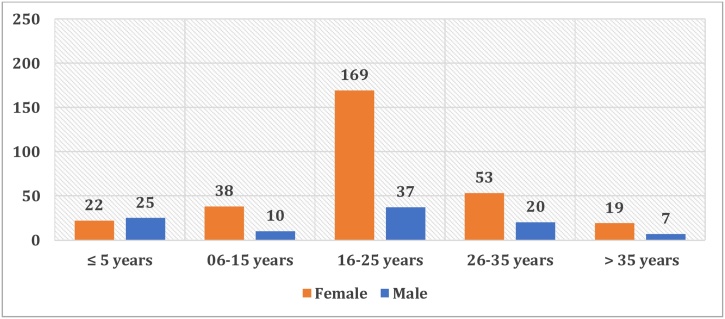
Distribution by age range and gender.

The average age of intoxicated is 20 ± 10-year-old (ages ranged from 4 months to 70-year-old). The range of teenagers (16 to 25-year-old) has a majority, followed by that of young adults (26 to 35-year-old). Together, these two ranges represent 70 % of the total number of cases (Fig. 2).

The incidence of acute Paracetamol poisoning in the child population (age ≤ 15) was about a quarter of the total. This frequency could be due to the curiosity of toddlers (age ≤ 5) and the lack of awareness of even schoolchildren (age 6–15) against the dangers of drugs.

The female gender remains the most affected (75 %). Women usually resort to drug poisoning to arouse attention and empathy, unlike men who, in attempts to autolysis, use generally more radical procedures. Eight pregnant women resorted to a voluntary intake of an important dose of Paracetamol especially for an abortive purpose. We quote the example of this 29-year-old woman, admitted to the emergency department of Oran University Hospital for the ingestion of 72 g of Paracetamol, following an unwanted pregnancy (4 weeks). The patient was conscious on admission with a blood pressure of 11/05 mmHg and a heart rate of 111 beats per minute. Hepatic and renal tests returned normal, and blood glucose at 0.96 g/L. A blood sample was received at the Department of Pharmacology Toxicology. Her serum paracetamol concentration was 129.6 mg/L at 13 h post-ingestion, thus indicating a high risk of liver damage according to the Rumack-Matthew nomogram. The patient received antidotal treatment (N-acetylcysteine) and she was discharge in good condition after 4 days of hospitalization.

Concerning the circumstances of the intoxication, these are suicide attempts in 82 % of cases, observed especially in adolescents (69 %). Depression, family and school difficulties, dependence on psychoactive substances, chronic diseases and violence are the most important reasons for suicidal acts among adolescents.

Accidental exposures occured in 12 % of cases and are exclusive to children. They constitute according to Bourrillon et al. the second cause of childhood accidents after traumas and before burns [1]. The age range of 1–5 years alone accounts for 89 % of accidental exposures in our study. This could be explained by the fact that the child at this age acquires a motor autonomy to satisfy his degree of curiosity, gradually discovering the environment, exploring the world around him and bringing everything he finds to his proximity, to his mouth [2]. Moreover, The sweet taste of Paracetamol syrup resembling that of sweets provides a pleasure to the child who does not hesitate to drink. The children also ingested oral suspensions, tablets and powders in dose sachet.

In 2 % of cases, this is a therapeutic overdose due in particular to concomitant use of several drugs containing Paracetamol (associated with other analgesics such as codeine and tramadol) or involuntary not-respecting the administration guidelines (doses, frequency of administration …) in order to relieve intense pain. Indeed, in the United States, unlike in the United Kingdom, half of the cases of overdose are apparently involuntary, with simultaneous consumption of many products containing Paracetamol for an analgesic effect [3].

Moreover, some factors could be the cause of an involuntary Paracetamol overdose even at therapeutic doses, including medical history such as: alcoholism, enzymatic inducing treatment including antiepileptic drugs (phenobarbital, phenytoin, carbamazepine), undernutrition, anorexia, HIV infection … etc. [4].

Furthermore, acute Paracetamol poisoning was suspected in some patients (4 %) who had suggestive symptoms, namely nausea, vomiting and abdominal aches evoking an eventual liver disorder. The toxicological analysis revealed a presence of Paracetamol in the blood in half of these patients.

Concerning ingested products, two-thirds of patients associated Paracetamol particularly with drugs (antidepressants, benzodiazepines and other analgesics). It’s clear that suicidal risk is often higher during the first few weeks of starting an antidepressant. All of the meta-analyzes concerning the presence of a link between antidepressant treatment and autolysis indicate an increase in suicidal risk in young people between 18 and 24 years old while a significant protective effect was found in 25–64 years old and even more among those over 65 [5].

**Fig. 3:**
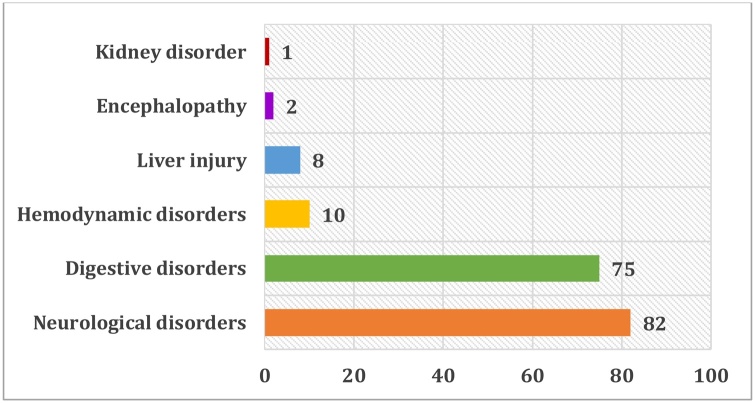
Distribution by clinical symptoms.

With regard to the symptoms of intoxication, half of the patients did not show any alarming sign because admitted in the first 24 h of intoxication which is an asymptomatic phase falsely reassuring. Neurological and digestive disorders were the most present (Fig. 3).

Neurological signs, such as drowsiness, dizziness, headache, and consciousness disorders, appeared in 82 patients, but this is not due to Paracetamol alone but due rather to the other associated drugs, particularly psychotropic drugs.

Digestive disorders were found in 75 patients (abdominal pains, nausea, vomiting). The clinical examination revealed a predominance of abdominal pain ranging from simple epigastric pain to pain in the right hypochondrium indicative of liver damage subsequently confirmed by a disturbed hepatic assessment.

The Paracetamol poisoning led to liver injury in 8 patients (abdominal pain in the right hypochondrium with jaundice or in the most severe forms, hepatic encephalopathy, hypoglycemia, hemostasis disorders leading to a hemorrhagic syndrome in a form of fulminant hepatitis).

As a reminder, the mechanism of acetaminophen-induced hepatotoxicity is as follows: Paracetamol (APAP or N-Acetyl-p-Aminophenol) has a high oral bioavailability (88 %); it is well absorbed and reaches the peak blood concentration within 90 min after ingestion. The liver and, to a lesser extent, the kidney and intestine are the major organs implicated in the metabolism of Paracetamol. After a therapeutic dose, Paracetamol is mostly converted to pharmacologically inactive glucuronide (52–57 %) and sulfate (30–44 %) conjugates, with a minor fraction being oxidized to a reactive metabolite N-acetyl-para-benzoquinoneimine NAPQI (5–10 %). NAPQI is highly reactive and is primarily responsible for Paracetamol-induced hepatotoxicity.

At therapeutic doses, detoxification of NAPQI occurs through its binding to the sulfhydryl group of glutathione (GSH) to form APAP-GSH, which is ultimately excreted in the urine [6]. However, after a highly toxic dose of paracetamol, once glutathione is depleted, NAPQI binds directly to cell proteins via cysteine residues, disrupts cellular integrity yielding hepatocyte necrosis. This injury likely takes place very rapidly once glutathione depletion is accomplished, leading to the extraordinary levels of aminotransferases [7].

Oxidative stress and inflammatory reactions were also incriminated in overdoses of Paracetamol inducing renal toxicity [8].

### 3.2 Toxicological analysis

**Fig. 4:**
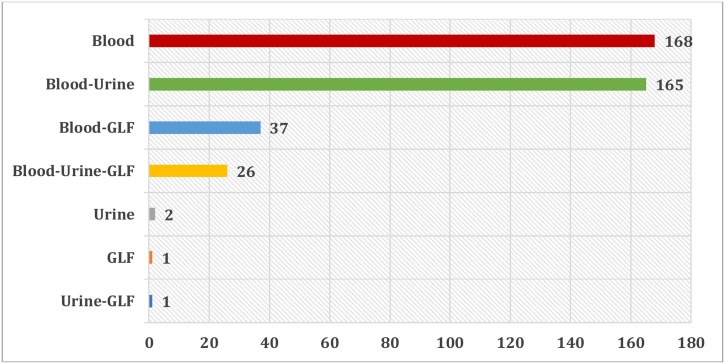
The biological samples received for the analysis of Paracetamol.

Among the 400 requests for toxicological testing, blood is the most frequently sample taken from patients and sent for toxicological assay (99 %) followed by urine (48.5 %) and gastric lavage fluid (GLF) (16 %) on which a simple qualitative research can be carried out (Fig. 4).

The interest of the determination of the serum Paracetamol level lies in the fact that it allows the estimation of the probability of hepatotoxicity and thus an adequate therapeutic management.

Urine is an interesting biological medium in addition to blood. Its analysis provides information on Paracetamol use over the previous 24–48 hours because of the rapid elimination of Paracetamol.

Gastric lavage fluid is the least retrieved sample because gastric lavage was performed for only a limited number of patients. It is also an important matrix for the qualitative research of Paracetamol, as much as the gastric lavage is carried out rather quickly after intake.

In the event of any admitted or suspected intoxication with Paracetamol, a toxicological testing systematically launched as soon as the doctor's request is received. It consists of a qualitative research on urine and/or gastric lavage fluid, a quantitative determination on blood or both.

Quantitative analysis on blood is essential. In addition to its diagnostic interest - which comes to comfort the qualitative research - it finds a utility in the evaluation of the gravity of the intoxication through the Rumack-Matthew nomogram and beyond the therapeutic interest by the indication of the treatment antidote based on N-acetylcysteine.

The toxicological analysis was carried out in 305 patients (76 %) including 247 quantitative determinations of Paracetamol on blood. This assay was not performed in some situations such as: non-compliant sample, device under servicing, shortage of reagents, etc.

**Fig. 5:**
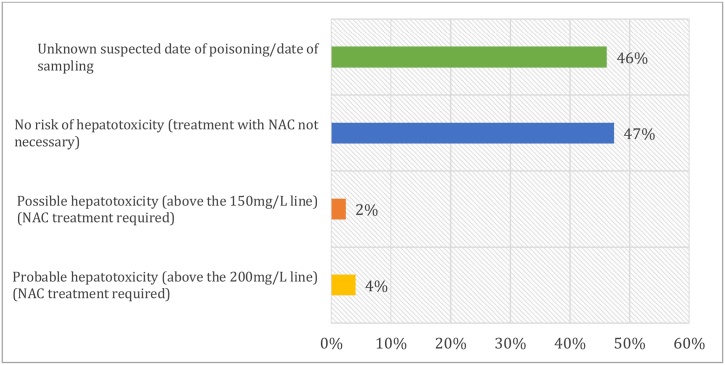
Interpretation of Paracetamol levels in the blood.

Taking into account the time elapsed since taking Paracetamol at the time of sampling according to Rumack-Matthew nomogram, these assays showed that 117 patients had values that were below the line defining the need for treatment while 16 patients had a high risk of developing liver injury and required antidote therapy (Fig. 5).

We take the example of this 17-year-old girl with a psychiatric disorder, admitted to medical emergencies for a voluntary drug poisoning by 16 g of Paracetamol with epigastric pain and vomiting.

The assay of Paracetamol on a blood sample taken 5 ½ hours after ingestion gives a rate of 195.4 mg/L which means, if we interpret on the Rumack-Matthew nomogram, a high risk of probable liver injury hence the indication of N-acetylcysteine administered at a loading dose of 150 mg/Kg/h followed by a maintenance dose of 100 mg/Kg/4 h than 50 mg/Kg/16 h. A second assay of Paracetamol in blood returned to 2.5 mg/L on a control sample done at 48 h ; antidotal treatment was stopped and the evolution was favorable.

Risk assessment of Paracetamol poisoning includes knowledge of either the ingested dose or the Paracetamol concentration and time since ingestion for patients who present early. Transmaninase measurement may contribute to the risk assessment in patients who present after first day [9]. The increase of transaminases occurs usually 24 h after ingestion of a toxic dose of Paracetamol [10].

In our study, the transaminase assay was requested for 50 patients. Canadian guidelines recommend the elaboration of this assay in the presence of any suspicion of Paracetamol poisoning [11]. The UK recommendations require that it be carried out only when the dose supposed to be ingested is greater than 75 mg/Kg. In situations where there is acute intoxication with unknown time of ingestion, transaminase disruption is a decision-making element of treatment initiation. In international recommendations, transaminases are checked again only if NAC is established and maintained in order to decide whether to continue or stop treatment with NAC [12].

Prothrombin Ratio, INR, and factor V reflect the severity of liver damage, but their variation occurs late. They were only requested in 2 cases in our study.

We report here the case of a patient aged 15 years admitted to the emergencies of the Oran University Hospital with dizziness, vomiting and abdominal pain. The patient admitted during the interrogation the taking of 38 tablets of a drug containing Paracetamol and Codeine. A toxicological assay performed at 5h15 post-ingestion revealed a serum Paracetamol concentration equal to 107.3 mg/L. This value according to the Rumack-Matthew nomogram does not represent any risk of liver toxicity, unlike the second assay performed on a sample taken at 48 h and which gave a serum Paracetamol level of 6.7 mg/L. That day, the hepatic assessment was disturbed indicating a rate equal to 169IU/L for the ALAT and 91IU/L for the ASAT. The patient underwent digestive decontamination with activated charcoal and received an antidotic treatment based on N-acetylcysteine. She was discharged in good condition after 3 days of hospitalization.

### 3.3 Therapeutic management and evolution

Different treatments have been introduced into our patients, taking into account the entire context of each patient's intoxication, namely a symptomatic, an evacuating and a specific treatment (N-acetylcysteine). No patient required the use of a dialysis treatment in our study.

Almost all patients received symptomatic treatment with standard monitoring, rehydration and administration of gastric demulcent in cases of poisoning where paracetamol is combined by the patient with anti-inflammatory drugs or antibiotics. Glucose serum is administrated in case of severe hypoglycemia. Indeed, it is very important to monitor blood glucose very regularly in case of severe overdoses and to correct it because during acute liver failure, there is a dysfunction of glycogenolysis and glyconeogenesis resulting in insulin resistance [13].

Gastric lavage was performed in 115 patients, or 28.75 %. It has been envisaged in cases of important ingestion of Paracetamol when the patient comes early enough (60 min post-ingestion). 211 patients, or 52.75 %, received activated charcoal. Indeed, a digestive decontamination by administration of activated charcoal is recommended within 2–4 hours following the ingestion of a toxic dose of Paracetamol in conscious and cooperating adults and in the absence of vomiting [14]. This decontamination would reduce serum Paracetamol concentration and the risk of liver toxicity [15]. In our study, gastric lavage was followed by the administration of activated charcoal in 35 intoxicated patients. According to the study by Kulig et al., This association within one hour of ingestion of the toxicant has shown clinical improvement in patients with impaired consciousness [16].

The key to successful management of a Paracetamol-poisoned patient is the rapid initiation of N-acetylcysteine therapy after diagnosis, which was performed in 77 patients in our study.

Three schemes of administration are known for N-acetylcysteine in the treatment of acute Paracetamol poisoning (Table 1). The scheme for oral administration is now standard in the United States, while in Europe it is intravenous administration that predominates. It is indicated from a Paracetamolemia serum Paracetamol concentration threshold at H4 of ingestion at 150 mg/L in France [9] and recently at 100 mg/L in the United Kingdom [17].

**Table 1:** Schemes of N-acetylcysteine administration.

Scheme of administration	Loading dose	Maintenance dose	Total dose	Total duration	Reference
Venous route (Prescott)	150 mg/Kg (60 min)	50 mg/Kg (4 h) then 100 mg/Kg (16 h)	300 mg/Kg	21 h	[18]
Venous route (Smilkstein)	140 mg/Kg	70 mg/Kg every 4 h, to repeat 12 times	980 mg/Kg	48 h	[19]
Oral route (Rumack)	140 mg/Kg	70 mg/Kg every 4 h, to repeat 17 times	1330 mg/Kg	68 h	[20]

All three schemes are equally effective if treatment is initiated within ten hours of ingestion of Paracetamol. In cases where treatment is initiated after more than ten hours, the oral administration scheme and the Smilkstein's intravenous administration scheme were better than with Prescott. N-acetylcysteine remains effective to a lesser degree if treatment is initiated more than 15 h after ingestion of Paracetamol [21].

In our study, the antidotic treatment was administered in 77 patients according to the Rhumack protocol. The administration of N-acetylcysteine was thereby per os because of the unavailability of the injectable form in Algeria.

Concomitant administration of activated charcoal and oral NAC was performed for 31 patients in our population. Renzi et al. have shown that charcoal does not alter the kinetic parameters of N-acetylcysteine thus confirming previous observations [22,23]. However, this association has been controversial by some authors because N-acetylcysteine is complexed by charcoal [24]. The latter will reduce the bioavailability of N-acetylcysteine from 29 % to 39 %. Some authors have proposed to avoid this association or to increase the N-acetylcysteine dose by 40 % - which is poorly tolerated by patients on the digestive plan - or to maintain an interval of 2 h between administration of N-acetylcysteine and activated charcoal. This problem can be avoided when N-acetylcysteine is administered intravenously [20,23].

In our study, one-third of patients stayed in hospital for one day. The duration of hospitalization was 48 h for 59 patients and 72 h for 26 patients. The length of stay was shortened to a few hours for 54 patients given the low dose supposedly ingested by the patient with no clinical symptoms or a non-alarming blood level of Paracetamol. The clinical condition of 21 patients required hospitalization for 4–7 days or longer. The latter were admitted to emergencies for poly-drug volunteer intoxication and presented with severe symptoms (mydriasis, revulsion of the eyeball, epileptic convulsive episode, hypotension, etc.).

The evolution was favorable for 336 patients, i.e. 84 % of the cases. In our series, 8 cases of liver injury were recorded as well as 5 deaths, i.e. 1.25 % of all acute paracetamol poisoning (Table 2).

**Table 2:** Information concerning patients with liver injury.

Gender, age, circumstances	Paracetamol taken dose	Paracetamol serum concentration	Clinical signs	AST/ALT rates	Treatment	Evolution
Female, 15 year-old, voluntary poisoning by paracetamol/codeine	38 g	107.3 mg/L at 5 h post-ingestion. 6.7 mg/L at 48 h post-ingestion	Dizziness, vomiting and abdominal pain	AST : 91 IU/L ALT : 169 IU/L	Activated charcoal, oral NAC	Favorable
Female, 26 months, accidental exposure to paracetamol	NIP	2.4 mg/L on day 10 post-ingestion	Altered state of consciousness, hepatomegaly on palpation, sub-jaundice, hematemesis, hypoglycemia, hepatic encephalopathy, convulsions	100 x ULN	Ventilation, fentanyl, diazepam	Died after cardiac arrest
Female, 2 year-old, accidental exposure of paracetamol and amoxicillin	1.5 g	0.6 mg/L at 2 h post-ingestion	Liver injury	4 x ULN	Oral NAC	Favorable
Male, 26 year-old, hypertensive patient, suspicion of paracetamol poisoning	NIP	0.8 mg/L (delay post-ingestion not precised)	Edema, palpebral epistaxis, coma post-anoxic following a cardioresp arrest, fever, suspicious hepatic encephalithy	NIP	Gastric lavage, hypnovel, fentanyl	Died few days after admission
Male, 34 year-old, poly-drug voluntary poisoning : risperidone, ibuprofen, paracetamol/triprolidine/pseudoephedrine, levomepromazine, Nebivolol, etc.)	NIP	8.9 mg/L (delay post-ingestion not precised) Presence of amitriptyline and phenothiazines in urine	Cyanose, coma carus, collapse	NIP	Not received	Died after cardiac arrest
Pregnant female, 20 year-old, voluntary poisoning by paracetamol, anti-inflammatory, haloperidol, bleach cleaning solution	NIP	The dosage of paracetamol in serum has not been done	Agitation, polypnea, blurred vision, hearing loss, hypotonic diuresis	NIP	NIP	Mydriasis, hemorrhage and convulsions then Death
Male, 28 year-old, HIV positive, suspicion of paracetamol poisoning	NIP	Absence of paracetamol in serum	Agitations, hepatic encephalopathy, acute liver failure	NIP	Not received	Died 3 days after admission
Female, 10 year-old, therapeuticoverdose *o*f paracetamol and acetylsalycilic acid	300 mg every 4 h for 2 days	The dosage of paracetamol in serum has not been done. Presence of paracetamol and acetylsalycilic acid in urine	Hematemesis, hemorrhagic hepatitis	NIP	NIP	Favorable

***NIP:****No Informations Provided.*

## 4 Conclusion

Although it seems benign, acute Paracetamol poisoning is serious and requires adequate care. It must never be trivialized.

The general population must be made aware of the dangers of Paracetamol. While dispensing drugs, the Pharmacist should provide informations about the recommended dose and the toxicity. Patient should read the safety inserts that come with medication, keep careful of which dose they are taking and store medicines safely away from children.

The quantitative determination of Paracetamol is a key element in assessing the severity of intoxication, predicting its prognosis and indicating the need for antidote administration. This requires collaboration between clinician and analyst.

## Funding

This work was supported by the General Directorate of Scientific Research and Technological Development, of the Minister of Higher Education and Scientific Research of Algeria.

## CRediT authorship contribution statement

**Bilel Chefirat:** Conceptualization, Methodology, Validation, Formal analysis, Investigation, Writing - original draft, Writing - review & editing, Visualization, Supervision, Project administration. **Anissa Zergui:** Conceptualization, Methodology, Validation, Formal analysis, Investigation, Writing - original draft, Writing - review & editing, Visualization, Supervision. **Chaïmaa Rahmani:** Formal analysis, Investigation, Writing - original draft. **Meriem Nour Belmessabih:** Formal analysis, Investigation, Writing - original draft. **Haciba Rezk-kallah:** Resources.

## Declaration of Competing Interest

The authors report no declarations of interest.
